# Elevation of IgE in patients with psoriasis: Is it a paradoxical phenomenon?

**DOI:** 10.3389/fmed.2022.1007892

**Published:** 2022-10-13

**Authors:** Leyao Shi, Chen Liu, Huabao Xiong, Dongmei Shi

**Affiliations:** ^1^The Second Clinical Medical College, Shandong University of Traditional Chinese Medicine, Jinan, China; ^2^The Laboratory of Medical Mycology, Jining No. 1 People's Hospital, Jining, China; ^3^Basic Medical School, Institute of Immunology and Molecular Medicine, Jining Medical University, Jining, China; ^4^Department of Dermatology, Jining No.1 People's Hospital, Jining, China

**Keywords:** Immunoglobulin E, atopic dermatitis, psoriasis, T helper 2 cell, T helper 17 cell

## Abstract

Immunoglobulin E (IgE) elevation is a hallmark of allergic conditions such as atopic dermatitis (AD). The pathogenesis of AD is typically associated with high levels of IL-4 and IL-13 produced by activated T helper 2 (Th2) cells. Psoriasis, on the other hand, is an inflammatory skin disease mainly driven by Th17 cells and their related cytokines. Although the immunopathologic reactions and clinical manifestations are often easily distinguished in the two skin conditions, patients with psoriasis may sometimes exhibit AD-like manifestations, such as elevated IgE and persistent pruritic lesions. Given the fact that the effective T cells have great plasticity to re-differentiate in response to innate and environmental factors, this unusual skin condition could be a consequence of a cross-reaction between distinct arms of T-cell and humoral immunity. Here we review the literature concerning the roles of IgE in the development of AD and psoriasis, showing that elevated IgE seems to be an important indicator for this non-typical psoriasis.

## Introduction

Immunoglobulin E (IgE) elevation is a useful clinical indicator for atopic dermatitis (AD) and other allergic diseases. Antigen-specific IgE *via* binding IgE receptors on master cells and basophils plays a central role in the initiation of mediator release from these cells, which often leads to immediate hypersensitivity reactions such as systemic anaphylaxis, bronchospasm, and urticaria. However, elevated IgE is also found in some chronic inflammatory allergic diseases such as rhinitis, asthma and AD (1, 2), and even non-allergic diseases such as psoriasis (3).

AD causes dry, itchy and inflamed skin lesions. Psoriasis is also a chronic inflammatory skin disease with patches of rashes and abundant silvery scales. Both conditions tend to be long-lasting and flare up from time to time. In contrast to the relatively young ages of onset in AD patients, psoriasis is common in adults. The primary immune cell effectors in the two diseases are believed to be different (4, 5). Many studies have shown that the elevated IgE and persistent pruritus in AD patients are driven by activated Th2 immune response (6–8). By contrast, pruritus in psoriasis patients is often mild or even absent (9, 10), and the immune mechanism of psoriasis appears to be activated by Th17 cells and is associated with high levels of IL-17A and IL-17F (11). However, when psoriasis patients exhibit severe pruritus as well as high levels of eosinophils and IgE (12–14), it remains unknown whether these conditions are comorbid characteristics of AD and psoriasis or simply manifestations of a kinetic spectrum of the individual diseases. In this review, we will focus on the involvement of IgE in the pathogenesis of both AD and psoriasis, and examine the possible IgE-mediated pathogenic mechanisms in AD and psoriasis.

## Biological functions of IgE

IgE is one of the five immunoglobulins. It consists of two heavy chains (H chains) and two light chains (L chains), which are linked *via* disulfide bonds to form a tetrapeptide chain molecule similar to other monomer immunoglobulin molecules (15). Structurally, IgE molecule has no hinge region that differs from IgG, IgA, and IgD (16). The main function of antigen specific IgE is to induce release of mediator molecules from mast cells or basophils after binding to the high-affinity IgE receptor (FcεRI) on these two types of immune cells (17). The mediator molecules such as histamine, prostaglandins, and leukotriene 2 will then enormously increase vascular permeability, bronchospasm, and others (18, 19). Mast cells and basophils are both derived from CD34^+^ hematopoietic progenitors. Mast cells are long-lived resident cells in tissues that differentiate from other blood-derived progenitor cells (20). Mast cells are found in areas that are often exposed to the external environment and microbiota, such as mucosa of the gastrointestinal tract and respiratory tract (21). Basophils, once mature in the bone marrow, enter the blood circulation and then are activated in blood vessels and transported to inflammatory sites to perform functions similar to the mast cells (22–24).

The Th2 immune response plays a central role in activating IgE-mediated activation of mast cells and basophils (25, 26). In addition to those effector molecules that are released from mast cells and basophils to cause clinical symptoms, the mediator molecules also include growth factors, cytokines and chemokines. Of them, the cytokines such as IL-4 and IL-13 are required for priming both Th2 cell differentiation and aiding the switching of B cells to IgE-producing plasma cells (27–29). Interestingly, IL-22 is highly produced by activated mast cells within the plaque lesions of psoriasis and AD. The immunopathogenic roles of IL-22 have been well established in the development of both diseases (30). When the allergic host is re-exposed, the allergen will cross-link with IgE already bound to the membrane and activate mast cell or basophils to release allergic mediators. This cascade of reactions culminates in the typical symptoms of type I hypersensitivity (31).

New evidence has suggested that the biological function of IgE is beyond the Type I hypersensitivity reactions. A number of studies have proven that IgE alone can provide survival signals to mast cells even in the absence of allergens (32, 33) or survival cytokines (34). Like normal antigen-binding IgEs, the so-called cytokinergic IgEs could bind FcεRI receptors on the surface of mast cells. However, this cross-linking stabilizes FcεRI receptors, which then prevents the degradation process of mediator cells (35). IgE has even been found to be involved in the antigen-presenting process (36, 37). Indeed, in terms of the magnitude of T cell activation, the antigen-IgE complex is several folds stronger than either the antigen-IgG complex or antigen alone (37). This process depends on the endocytosis of the antigen-IgE complex through CD23 expressed on B cells or FcεRI on dendritic cells (DC), which is named IgE-facilitated antigen presentation (FAP) (38). The FAP mechanism is thought to be presented in most allergic and atopic diseases, and is also responsible for the relapse of atopic diseases.

Despite the short half-life of IgE, sustained IgE production appears important to maintain allergen-specific IgE levels in allergic patients, most likely due to long-lived plasma cells and memory cells (39, 40). IgE-producing plasma cells and IgE^+^ memory cells have been detected in the blood of allergic patients, but these cells produce only about 0.2% of IgE in serum because of few such cells in the blood. Therefore, it is likely that most IgE-producing and IgE memory cells may exist elsewhere (41). For instance, lymphoid tissues at sites of allergen exposure (e.g., nasal mucosa and intestinal mucosa) contain IgE^+^ memory cells that can be activated (42) or plasma cells in the bone marrow (43, 44).

Elevated IgE has been associated with the severity of patients with exogenous AD and normal IgE levels have been found in patients with intrinsic AD (45, 46). Recently, IgE autoreactivity that targets keratinocytes and a variety of autoantigens has increasingly received attention in research of AD immunopathogenesis (47–49). These IgE autoantibodies also play an important role in exacerbating and prolonging the severity of AD. By contrast, when paradoxical eczema appears in patients with psoriasis, the IgE elevation tends to vary with stages of psoriasis (50) and biological drug treatments the patients received (51, 52).

## Immunopathological relationship between atopic dermatitis and IgE

### Pathogenesis of atopic dermatitis

Failure to maintain the skin barrier could initiate the onset of an atopic condition such as AD (53). Genetic defects in AD patients are mainly involved in mutations of the filaggrin (FLG) gene (54–57). Some FLG mutations would affect the development of the stratum corneum and change pH on the skin surface (58). As a result, the thymic stromal lymphopoietin (TSLP) is produced by the barrier-disrupted epidermis, which effectively induces a Th2/Th22 immune response in AD lesions (6, 59). Increased IL-4/IL-13/IL-25 and IL-22 form a positive feedback loop that further suppresses FLG expression in the stratum corneum barrier (60) and increases allergen penetration and systemic IgE sensitization (61).

### IgE mediated type 2 inflammatory reaction in atopic dermatitis

Skin barrier defects and atopic immune environments work together to promote the development of a series of IgE-mediated allergic diseases, known as atopic march, in a sensitized patient (62). The highly produced IgE tends to skew the adaptive response toward type 2 inflammatory reaction diseases in each of these atopic conditions (6). Serum IgE levels are elevated in about 80% of AD patients sensitized to air and food allergens (7). Even less common, AD patients can be caused by the excessive autoantibody reactive to IgE. In this case, auto-reactive IgE antibodies induce an allergic, autoimmune process that tends to be persistent and chronic inflammation course (63). Meanwhile, the increased sensitivity to autoantigens observed in AD patients may concur with other autoimmune diseases such as systemic lupus erythematosus and rheumatoid arthritis (63–65).

## Potential association between psoriasis and IgE

### Pathogenesis of psoriasis

The immune effectors of psoriasis include Th1, Th17, and Th22 and their corresponding cytokines such as IFN-γ, IL-17, and IL-22 (5, 66, 67). At the onset of psoriasis, the cytokines secreted by Th17, keratinocytes and other skin-resident cells, could work together to induce an extensive inflammatory response and recruit Th1 and Th22 cell subsets into psoriatic lesions. These effector cells then promote the cytokine secretion to magnitude inflammatory responses. This process has been known as “feed-forward” in the pathogenesis of psoriasis (11, 68). Of note, clinical studies demonstrate that antagonists of IL-17 alone are sufficient to remiss the disease (69, 70). Thus, it is believed that Th17 cells are the main driver of psoriasis pathogenesis.

Keratinocytes play an essential role in psoriasis. Following the activation, keratinocytes indirectly attract immune cells into the skin by releasing chemokines, such as CXCL8, which is the major recruiter for neutrophil accumulation in the epidermis (71). Other keratinocyte-produced chemokines, such as CCL2, CCL5, CXCL10, and CXCR3 ligands, participate in attracting monocytes and Th1 cells (72). In addition, ADAMTSL5, highly expressed in keratinocytes, can act as an autoantigen in psoriasis (73). When activation of psoriatic Th17 cells is not driven by exogenous antigens in psoriasis, chronic activation of endogenous autoantigens could be the alternative mechanism for inflammatory reactions due to insufficient Tregs and immune checkpoints responses (74). Because Th17 cells can promote the production of both ADAMTSL5 and another skin autoantigens, LL37, the such positive feedback loop may exacerbate the course and chronicity of this disease (75). Finally, activated keratinocytes and immune cells would release endothelial growth factor that increases capillary density and permeability in psoriatic lesions.

### IgE in psoriasis

The presence or absence of a Th2 immune response is the discretionary sign to discriminate psoriasis from AD. However, accumulating evidence shows that some psoriasis patients have high levels of serum IgE and tend to have pruritic AD-like manifestations (3, 12, 76, 77). It seems that serum IgE levels are higher in patients with more severe psoriasis (3) or with longer skin lesions (50). A study in Austria divided patients with psoriasis into mild (PASI < 10) and moderate-to-severe (PASI ≥10) groups and found that the allergic conditions appeared in 29.0% of patients in the moderate-to-severe group and 23.8% in the mild psoriasis group, although Type I hypersensitivity reactions in both groups were similar to the general population (12). In terms of the IgE level, 34.7% of the psoriasis patients showed elevated IgE in the PASI ≥ 10 group compared with 19.1% in patients with PASI < 10 (78).

IgE levels in patients with psoriasis vary with stages and types of disease. A radio allegro sorbent test (RAST) is a commonly used method to measure the serum IgE in the clinical setting. A previous study showed that RAST was positive in 58 and 22% of patients with chronic plaque psoriasis and active psoriasis, respectively (79). Several studies showed similar results, in which the positive RAST tests were significantly higher in patients with chronic plaque psoriasis(CPP) than those with progressive psoriasis (50, 80). However, contradictory results have also been reported. For example, the proportion of patients with elevated serum IgE in psoriasis vulgaris (PV) group was 77%, which was significantly higher than that in CPP group (29.7%) (81). For PV and generalized pustular psoriasis (GPP), the percentage of patients with an elevated IgE accounted for 46 and 76.2%, respectively, compared with 15.6% in healthy controls (82). Compared with 76.5% of patients with psoriatic erythroderma (PE) that showed elevated IgE, only 37.9% CPP patients showed high IgE (50). Similarly, another study found that 81.3% of PE patients had elevated serum IgE, which was significantly higher than that of controls (6.3%) (83). All these studies suggested that PE patients are more likely to have high serum IgE and that patients with moderate-to-severe psoriasis with joint symptoms have higher serum IgE levels than patients without joint symptoms (84).

The pathological impacts of elevated IgE may differ in different forms of psoriasis that may determine the clinical outcomes. Patients with a longer period of skin lesions had higher total IgE concentrations (50), and serum IgE levels correlated positively with C-reactive protein (CRP) in patients with GPP and PV (82). In addition, the populations of IgE^+^ cells and FcεRI^+^ cells are substantially higher in psoriatic lesions relative to non-lesional areas of the skin in the same patient, suggesting that FcεRI^+^ cells may also be involved in the development of psoriasis (85, 86). However, there are also contradictory result. In one study, high serum IgE in patients with psoriatic arthritis (PsA) seemed to suffer fewer episodes of atopic symptoms when compared with PV and control groups (87), suggesting that atopic disorders somehow protect against the development of PsA. Results from several studies may provide a clue to explain the significance of the elevated IgE in PsA and PE patients. First, the elevated serum IgE levels in PsA seem to be a consequence of an altered Th1/Th2 balance (84); the Th1 / Th2 response was skewed toward the Th2 axis in PE patients (83). Th1 lymphocytes may produce cytokines in the early phase of psoriasis, whereas Th2 lymphocytes cytokines for activating IgE produce appear at the late stage of psoriasis. Therefore, as others have suggested, caution should be exercised in using Th2-inducing reagents when treating patients, particularly PsA and PE patients (81). Second, it is interesting to observe that the IgE elevation was correlated to a down-regulation of CD23 in B cells harvested from synovial fluid from PsA patients (88). CD23 is a low-affinity IgE receptor. How this down-regulated CD23 affects IgE-mediated inflammation in PsA is unclear.

Overproduction of IgE is mainly driven by Th2-associated cytokines, such as IL-4 and IL-13 (89). However, IL-17 also participates in inducing the differentiation of IgE-secreting cells and promoting the synthesis of IgE (90). Keratinocytes do not produce IL-4 or IL-13 but express IL-4 and IL-13 receptors on the surface of the cells (91). Given that the over-proliferation of keratinocytes and high production of IL-17 are common in psoriasis patients, one can imagine that both keratinocytes and IL-17 may contribute to the elevated serum IgE in psoriasis. For example, upregulated IL-21 cytokine has been noted in psoriasis, which has been speculated to be involved in IgE production in psoriasis (92, 93). Proinflammatory cytokines such as IL-6, IL-17, IL-21and CD40/CD40L may play critical roles in the elevation of IgE in psoriatic lesions (86, 94).

To date, a number of biologics have been used to effectively treat psoriasis, including psoriasis patients with high serum IgE levels. Ustekinumab downregulates IL-17 by binding promoters of IL-12 and IL-23 (IL-12 and IL-23 inhibitor), which can significantly reduce IgE^+^ and FcεRI^+^ cells in patients with high serum IgE levels. Since IL-17 has been shown to promote IgE production from human B cells, the downregulation of IL-17 by ustekinumab may thus decrease IgE (92). However, it is noteworthy that ustekinumab paradoxically initiated or exacerbated AD-like symptoms (95, 96). Risankizumab, an IL-23 inhibitor, was reported to cause the elevation of serum IgE in patients with psoriasis. Others reported that patients with plaque psoriasis developed an extensive urticaria rash with intense itching after 1 month of treatment with efalizumab (52). Taken together, allergies appear to be common in patients with psoriasis. These results suggest that allergen exposure should be avoided or combined with anti-allergy therapy, particularly in patients treated with biologicals (78). Nonbiological agents, such as azithromycin or methotrexate, can also decrease the serum IgE levels (86).

Despite accumulating evidence showing that an elevated level of total serum IgE is associated with psoriasis, the involvements of IgE in psoriasis pathogenesis are not fully clarified. The elevated IgE level in psoriasis patients suggests a possible shift of Th17 toward Th2 immune responses, which makes it difficult to distinguish between psoriasis and AD. Since the biologics for psoriasis treatment have a profound effect on modulating different subsets of T-helper cells, results from studies on the immunopathogenesis of psoriasis could become more complicated under biologic therapies (97).

## IgE is a link or epiphenomena for atopic dermatitis and psoriasis

### Overlap between psoriasis and atopic dermatitis

Traditionally, AD and psoriasis are considered two distinct diseases by virtue of their immunopathogenic mechanisms, clinical characteristics, and the ages of onset (98). However, when one views the dynamic process of each individual disease, some AD patients with high levels of Th1 and Th17 cytokines may mimic psoriasis and show psoriasis-like lesions. On the other hand, some psoriasis patients may present with AD-like lesions with high IgE and severe pruritus. The coexistence of psoriasis and AD includes different conditions such as psoriasis in patients with AD, psoriasis in children with AD, AD in children with psoriasis, contact dermatitis in psoriasis, and a phenotypic shift to eczema as a result of biologic therapy for psoriasis (99–104). Therefore, the coexistence ratio is also different (Table 1).

**Table 1:** The distribution of subtypes of Psoriasis and AD in the published articles.

**Subtypes**	**Percentage (%)**
Psoriasis in patients with AD (97, 99)	3–16.7%
Psoriasis in children with AD (98)	0.2%
AD in patients with psoriasis (97, 99)	2–9.5%
AD in children with psoriasis (98)	1%
Contact dermatitis in psoriasis (100, 101)	32.7–77.7%
Phenotypic shift to eczema as a result of biologic therapy for psoriasis (102)	6.0% (Infliximab) 2.7–12.1% (Ixekizumab) 3.9–8.0% (Secukinumab) 4.4% (Ustekinumab)

Common genetic predispositions have been noted (105) for the two diseases, in which the mutations are found on chromosomes 1Q21, 3Q21, 17Q25, and 20P12 (106). In addition, associations with AD were found on chromosomes 1Q21, 17q25, and 20P, regions that closely correspond to known psoriasis sites (107). A study in Japan found a marginal association between AD and two psoriasis susceptibility SNPs, IL-13(rs1295685) and ZMIZ1(rs1250546) (108). Although it is generally believed that the FLG gene mutation contained in the epidermal differentiation complex (EDC) on chromosome 1Q21.3 is more closely related to the development of AD than psoriasis (109, 110). Variants of the FLG mutation have been reported to increase the risk of psoriasis in Chinese patients (111). Interestingly, the two diseases had another common region on the genome, chromosome 5Q31.1-Q33.1, where IL-13 was associated with both AD and psoriasis (109). There is evidence supporting a significant correlation between IL-13 and psoriasis/ psoriatic arthritis (112, 113).

Clinical symptoms also overlap or coexist between the two diseases, which may present a challenge for clinical diagnosis. Studies have found that patients with overlapping AD and psoriasis have a high incidence of hand involvement (101). Because adult AD patients are more likely to appear on body flexion, the lesions on the hand often exhibit atypical manifestation (114, 115). As a large number of proinflammatory cytokines and chemokines are involved and released into the circulation in AD and psoriasis, the systemic inflammatory spectrum of clinical manifestations has also been described as “psoriatic march,” “march of psoriasis” or “inflammatory skin march” (116). Itching is one of the hallmark symptoms of AD and occurs in many psoriasis patients (117–119). The mediators causing this symptom include neuropeptide substance P, its receptor NK-1R, and β-endorphin precursor gene (POMC), which are upregulated, and a downregulated κ-opioid receptor gene (OPRK1). MRGPR, an enzyme of cytosolic group IV PLA2 family members and TRPV1 are also significantly elevated in pruritus skin (120). Studies have shown that upregulated phospholipase A2 IVD, Substance P, Nav1.7, or TRPV1 genes in itchy skin are positively correlated with the intensity of pruritus in AD and psoriasis. Also, cytokines such as IL-17A, IL-23A, and IL-31 are upregulated in atopic pruritus and psoriatic skin (120).

#### Atopic dermatitis presents with psoriasis-like features

AD can be categorized into the extrinsic and intrinsic types, in which exogenous AD is frequently associated with activation of Th2 and elevated IgE and intrinsic AD shows activation of Th17 response besides activation of Th2. In adults, extrinsic AD with a decreased Th17 response tends to have nonflexural area involvement (121). S100A9/12, regulated by IL-17 and IL-22, was also significantly increased in skin lesions in intrinsic AD and it was positively correlated with SCORAD scores (122). In the acute phase of AD, thymic stromal lymphopoietin (TSLP) produced by keratinocytes can trigger Th2 polarization to produce IL-4 and IL-13, which in return acts on keratinocytes to further increase TSLP levels. This process thus creates a positive feedback loop (6).

AD starts in early childhood but has a tendency to persist throughout life with different clinical manifestations (123, 124), which are the consequence of dominated immune responses. For example, the Th2 response is detectable in the blood of infantile AD patients and the Th17 response in skin lesions then develops with increasing age along with Th2 and Th22 responses (59). However, when comparing no age difference in the expression of Th2 and Th22, a more pronounced Th17 response can be clearly detected in childhood AD patients than in adulthood AD patients (125). This may be related to age-related changes in the skin immune system, the regulatory and protective properties of which tends to skew a strong Th17 response in early life (126).

The clinical manifestations of AD may vary considerably in AD patients of different ethnicities. Lesions in Asian AD patients are more likely covered by scales than in European and American patients, and epidermal hyperkeratosis and neutrophilic infiltration are more common in Asian AD patients than European and American patients (55, 124, 127, 128). In addition to exhibiting psoriasis-like features, Asian AD patients often show a robust Th17 response (IL-17A, IL-19 and S100A12) in the skin lesions (129) and are more susceptible to *S. aureus* colonization, perhaps due to a lack of AMPs, damage to the skin barrier and dysbiosis of the microbial ecology. In this case, not only can *S. aureus* directly disrupt skin barrier function, but it can also cause Th17 polarization by upregulating pro-inflammatory cytokines. This may explain why some AD patients have lesions that are more similar to psoriasis (130, 131). Taken together, pathological mechanisms at different stages of the disease and different ages of the patients all contribute to a more psoriasis-like presentation in AD patients.

#### Psoriasis mimics atopic dermatitis

The function of Th17 cells and IL-17A, the signature cytokine of Th17 cells, are pivotal for the development of psoriasis (68, 132). IL-17A is also involved in the induction of Th2 cell-specific allergen activation and plays an important role in increasing serum IgE levels (133). There is a large amount of literature showing that Th17 cells are directly or indirectly involved in the development of allergic atopic diseases (134, 135). Unlike AD, Th2 cell response was downregulated in psoriasis patients, showing ethnic differences. A strong Th2 response was detected in the lesions of Chinese patients (136). IL-17 family has five other members (IL-17B, IL-17C, IL-17D, IL-17E, and IL-17F), which may play similar or opposite roles of IL-17A in regulating immune responses, *via* perhaps the Th1/Th2 balance or the Th17/Th2 balance (137). Taken together, these studies suggest that different members of the IL-17 superfamily can play different roles that can change the balance of Th1/Th2, which is part of the reasons that two diseases occur in the same patient.

IL-4 is considered a marker of Th2 cells, but interestingly, high levels of IL-4 can be found in patients with mild PASI scores (138). Since IL-4 can exert an anti-inflammatory effect by reducing the expression levels of IL-1 and IL-6 in the skin of patients with psoriasis, this phenomenon may be the result of the immune system trying to balance the main inflammatory responses. Such a balancing process can be demonstrated in psoriasis patients receiving biologic treatment. A study found that biologics that inhibit the TNF-α and IL-17/IL-23 axis induce atopic eczema, eosinophilia and increase serum IgE (104). Naïve CD4^+^ T cells differentiate into different subpopulations of Th cells in response to different stimuli. Whether this inhibition of IL-17 skews the immune responses toward a specific Th identity, or repolarize into new Th cell subsets are poorly understood. However, studies have found that Th17 cells are plastic, and in the presence of appropriate signals, they also shift to the Th2 phenotype that will turn human circulating memory CD4^+^T cells into the Th cells that produce IL-17A and IL-4 (139, 140). A previous study re-evaluated the spectrum plasticity and found that several new cytokine patterns were produced after repolarization. Repolarization of Th17-trained cells revealed severe instability in this spectrum. These cells can re-differentiate to the Th2 phenotype not only in the context of Th2 stimulation but also in the presence of neutral conditions (Th0). Thus, nascent Th17 cells may change cytokine expression toward the Th2 pattern under specific changes in an inflammatory microenvironment caused by preconditions (141). This further suggests that progression of the disease is a dynamic process with many strands in a possible network.

The skin barrier acts as a physical barrier and immune barrier, and the destruction of the skin barrier is associated with the process of AD pathology and the development of psoriasis. In psoriasis, keratinocyte proliferation and differentiation can be interfered with mutations in keratin (142), as well as decreased expression of the epidermal differentiation markers loricrin and FLG (143, 144). Dysregulation of tight junction proteins and E-cadherin, as well as CX26 overexpression, can also affect intercellular junctions (145–148). In addition, disorders of ceramide subtypes and skin lesions result in cuticular extracellular matrix dysfunction, low expression of aquaporin-3 (AQP3), and fibroblasts in psoriasis, all contribute to the dysfunctional physical barrier (149–153). For the immune skin barrier, keratinocytes trigger psoriatic inflammation by producing autoantigens (154) and promote DC autoantigen recognition by producing polyamines that prevent autoantigen RNA degradation (155). In addition to further contributing to psoriatic inflammation by producing proinflammatory cytokines (156, 157), keratinocytes can also produce chemokines that induce immune cell infiltration into the dermis (158) and contribute to psoriatic inflammation by means of direct contact between keratinocytes and T cells (159). Innate immune cells, including dendritic cells, neutrophils, macrophages, and mast cells, also produce cytokines, particularly THF-α, IL-17, and IL-22 (160–164), thus having a similar effect in driving inflammatory response in psoriasis. Dysregulation of immunosuppressive cells is closely related to psoriasis pathology. Regulatory T cells (Treg) are the most characteristic immunosuppressive cells. Suppression of Tregs results in increased penetration of γδ, CD4+, and CD8+T cells, an increased presence of IL-17 and tumor necrosis factor-α, and increased severity of skin lesions (165). Regulatory B cells (Breg), which exert immunosuppressive effects by producing the anti-inflammatory cytokine IL-10, was reduced in the circulation of patients with psoriasis (166). Moreover, the anti-inflammatory effect of Myeloid suppressor cells (MDSCs) was decreased in patients with psoriasis (167).

Patients with psoriasis have elevated serum IL-31 levels, which are associated with the presence of pruritus in these patients (168, 169). Persistent scratching of the skin would further damage the skin barrier in patients and lead to Koebner's phenomenon (170). Scratching could activate sensory neurons, force them to release proinflammatory neuropeptides, and again exacerbate skin barrier disorders in psoriasis (171).

Previous studies have strongly linked changes in the skin barrier with the development of allergic reactions (172), and dysfunction of the epithelial barrier is thought to initiate a range of allergic inflammatory diseases, including asthma and atopic dermatitis (173–175). FLG is a key component of the cuticle, and its genetic defects are believed to be involved in the development of psoriasis and AD and other IgE sensitization (176). Thus, in the skin with a broken barrier, alterations in the epidermal microenvironment appear to be particularly suited for producing allergen-specific IgE and type 2 inflammatory diseases (177), which are observed in some psoriasis patients who develop AD-like lesions.

### IgE is a link or epiphenomena for psoriasis and atopic dermatitis

In mouse models, there are two types of IgE induced *via* natural and adaptive ways (178). Adaptive IgE is usually produced by B cells and plasma cells, with characteristics such as variable region gene polysomatic high mutation. Natural IgE, unlike adaptive IgE, is produced by B cells and does not require the co-stimulation of antigen selection and T cells. Therefore, B cells that naturally produce IgE have less polysomatic high mutation and can respond to their own antigens (179). Some studies have found that high IgE VH mutation and the increased CDR3 diversity along with high serum IgE content in AD and psoriasis patients (178). In addition, it was found that the serum IgE level of Sequestosome 1/ P62 DKO mice returned to be normal, which was increased in psoriatic lesions, suggesting that this gene has a similar IgE regulation mechanism in psoriasis and AD (180).

Some studies have found that the up-regulation of ICOS on TH22 cells may play an essential role in the pathogenesis of AD in Han Chinese (181). Activation of the Th22 pathway plays an important role in the pathologic process of both psoriasis and AD (182). The development and biological functions of T cell subsets are critically regulated by the Inducible T cell co-stimulator (ICOS) and ITS ligand (ICOSL). The number of ICOS^+^ Tfh cells was also found to be positively correlated with the course of psoriasis (183). Additionally, it has been shown that ICOS-mediated stimulation promotes B cell differentiation and IgE production (184). Thus, the elevated IgE levels may be related to this pathway.

In addition, DC and T cell markers (CD3, ITGAX/CD11c and CD83) were expressed at higher levels in AD and psoriasis tissues (185). The serum total IgE level and IgE^+^ and FcεRI^+^ cells were significantly increased in psoriatic skin lesions, which were mainly expressed on mast cells, dendritic cells and macrophages (86). However, it is unclear whether the initial high IgE level initiates the inflammatory process or whether the increased number of proinflammatory cells leads to increased IgE production. CD3^+^ cells are the main cell population infiltrating psoriatic plaques (30) and in both acute and chronic AD lesions (6). However, IgE synthesis requires homologous interactions between the TCR/CD3 complex on T cells and MHC class II antigens on B cells, as well as contributions from cell adhesion molecules (186). CD83 is a marker of B cell activation and is important for B cell activation. In a B cell-specific CD83 conditional knockout model, bacterial clearance was inhibited, and the model showed a characteristic transformation to a Th2 response produced by high IgE (187) and marked by IL-4 and IL-13 production (188). The massive release of thymic stromal lymphopoietin (TSLP) from the barrier-disrupted epidermis also triggers a Th2/Th22 immune response, as a major T-cell response often observed in AD lesions. Furthermore, IgE-antigen complexes promote antigen delivery and skew the adaptive response toward type 2 immunity, which results in chronic skin barrier dysfunction and inflammation.

**Figure 1:**
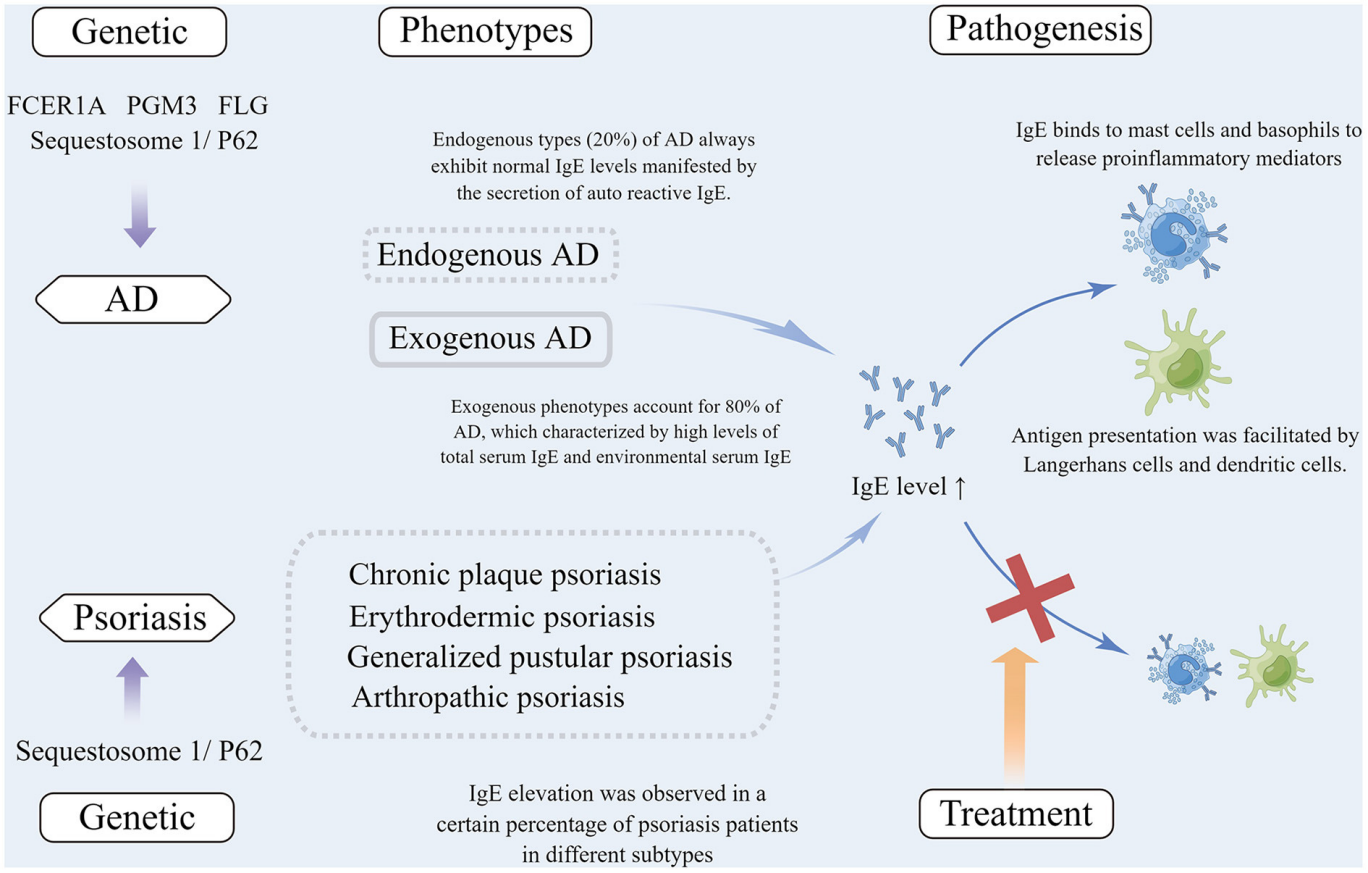
(1) Mutations of several genes are associated with IgE elevation in atopic dermatitis, including FCER1A, PGM3, FLG and Sequestosome 1/P62, among which Sequestosome 1/ P62 mutation was also observed in psoriasis patients with elevated IgE; (2) Exogenous phenotypes account for 80% of patients with AD, which are characterized by high total serum IgE and allergen-specific IgE. Patients with endogenous types (20%) of AD always exhibit normal IgE levels manifested by the secretion of autoreactive IgE. IgE elevation was observed in a certain percentage of patients with different subtypes of psoriasis; (3) In the pathogenesis of AD, IgE binds to immune and non-immune cells that expressed high-affinity IgE receptors (FcεRI) (e.g., dendritic cells, Langerhans cells, mast cells, basophils, and keratinocytes) to release proinflammatory mediators or to facilitate antigen presentation. In psoriasis, IgE can be bound to the above cells that expressed high-affinity receptors to be involved in the pathogenesis; (4) Anti-IgE treatment is often effective in AD patients, but there are no reports concerning anti-IgE treatment for psoriasis. AD, Atopic dermatitis; IgE, Immunoglobin E; FcεRI, Fc epsilon receptor Iα; PGM3, Phosphoglucomutase 3; FLG, Filaggrin.

The reciprocal regulation of Th1 and Th2 cells is always observed in psoriasis and other infectious diseases, with induction of Th1 cells and release of Th1-associated cytokines IFN-γ and TNF-α resulting in the inhibition of Th2 cells (189). Accordingly, it has been postulated that Th1-triggered diseases may be involved in the suppression of Th2 cells (190). Accumulating clinical data show that allergy appears to be equally abundant in patients with mild or moderate-to-severe psoriasis (78, 191). The genetic and phenotype, pathogenesis, and treatment of AD and psoriasis associated with elevated IgE levels were summarized in Figure 1.

### Potential causes of IgE stimulation in atopic dermatitis and psoriasis

An important mechanism for AD development is the disruption of the skin barrier that increases the transmissibility of external allergens and promotes the Th2 immune response through antigen-presenting cells such as Langerhans cells and dendritic cells, both of which could lead to increased IgE production (192). In addition, AD was found to have IgE autoantibodies and a wide range of autoantigenic epitopes (193–195). Autoreactive antibodies also seem likely to play a role in co-morbidities of AD and enhance atopic marching, as well as other inflammatory diseases (193). The presence of both exogenous and autologous allergens may then accelerate the immune response.

**Figure 2:**
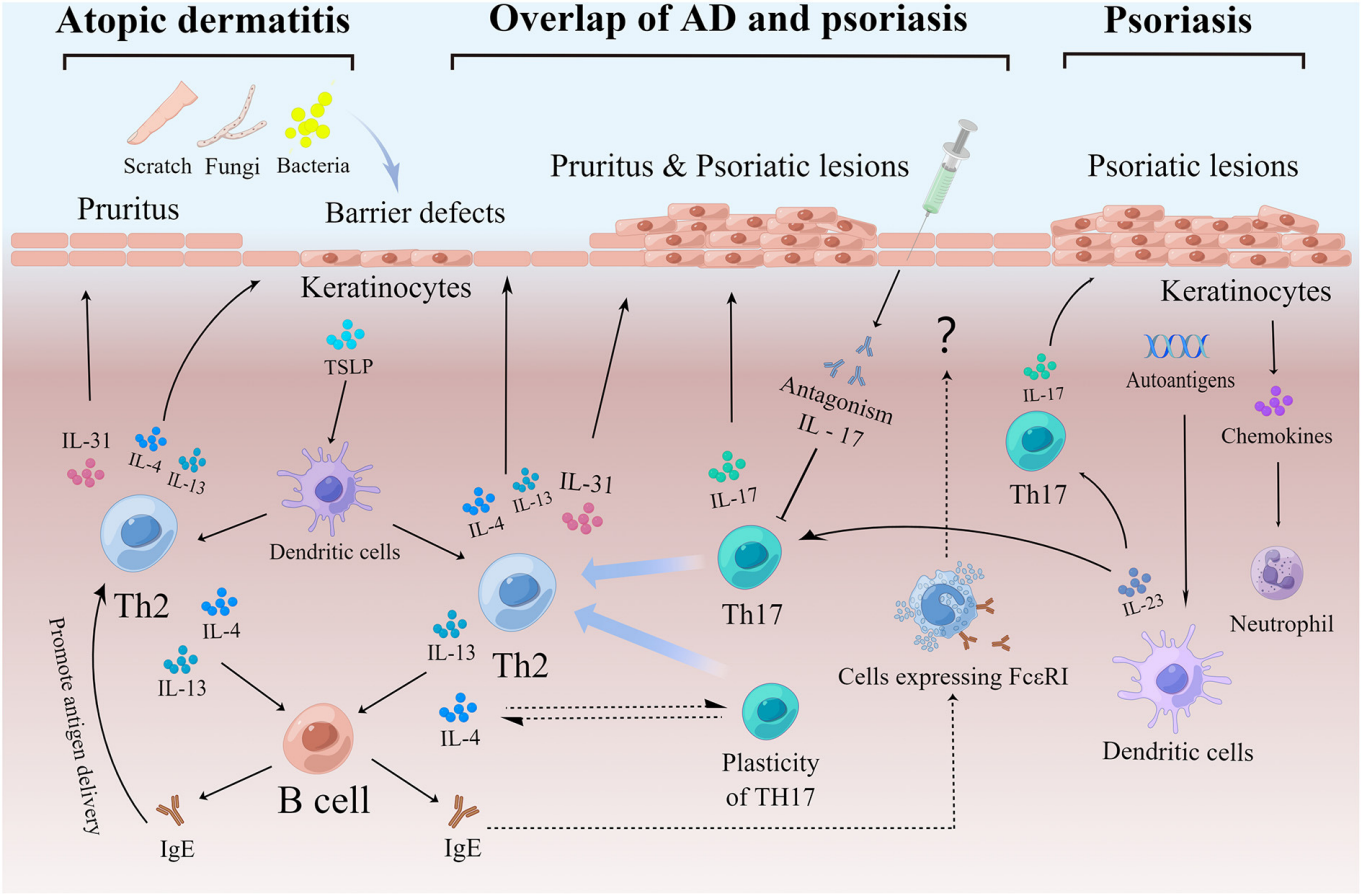
(1) An important pathogenesis of AD is the destruction of the skin barrier, which makes it easier for pathogens and allergens to invade and activate Th2. Keratinocytes play an important role in the inflammatory response to AD. Keratinocytes produce thymic stromal lymphopoietin (TSLP) to promote the differentiation of inflammatory Th2 cells. Th2 cells can produce cytokines, including IL-4, IL-13, and IL-31. IL-4 and IL-13 stimulate B cells, to produce IgE antibodies and aggravate skin barrier defects. IL-31 causes itching and stimulates scratching, which in turn damages the skin barrier. In addition, IgE promotes antigen presentation, which tilts the reaction toward Th2 and exacerbates the process. (2) Childhood AD patients, endogenous AD patients, Asian AD patients, etc. also show elevated Th17, thus exhibiting a partial overlap with psoriasis. In addition, certain percentage of psoriasis patients present with AD-like symptoms such as elevated serum IgE and pruritus, which may be due in part to Th17 remaining plastic and shifting to Th2 in response to stimulation mainly by IL-4. In addition, some patients with psoriasis show a shift to an eczematous phenotype following the use of IL-17 inhibitors, which may be due to a suppressed Th17 response and a relatively enhanced Th2 response. IgE produced during this process may act through cells expressing IgE high-affinity receptors. (3) Upon activating by a variety of stimuli, dendritic cells secrete IL-23 and further stimulate Th17 differentiation to produce IL-17. The IL-17/IL-23 axis plays a central role in the pathogenesis of psoriasis. Keratinocytes also play an important role in psoriasis by releasing chemokines to recruit neutrophils. In addition, keratinocytes with high expression of autoantigens may play a role in maintaining the pathological state of psoriasis. AD, Atopic dermatitis; IgE, Immunoglobin E; Th, T helper cells; DC, Dendritic cell; IL, Interleukin.

IgE is usually associated with type I hypersensitivity reactions and is not present in non-allergic diseases such as psoriasis. Although the roles of IgE in psoriasis pathogenesis remain unclear (188), the high levels of IgE in psoriasis (Figure 2) have become evident, as shown from numerous clinical and experimental studies of patients with chronic plaque psoriasis, erythrodermic psoriasis, generalized herpetic psoriasis, and psoriasis with comorbid arthropathy (50, 79, 80, 82–84). The data also show that serum IgE levels seem to be higher in patients with more severe psoriasis and with lesions of longer duration (3, 50, 78), highlighting the possibility that IgE could be a key indicator of AD-like psoriasis and play a potential role in the pathogenesis of AD and psoriasis. Based on these observations, psoriasis patients with AD-like symptoms and high IgE should avoid exposure to allergens and be aware of any lesion changes and possible shifts to eczema or urticaria (51, 52, 97) under biologic therapies.

## Conclusions and future perspectives

Although AD and psoriasis have traditionally been considered two clinically distinct diseases, it has become clear that overlapping clinical manifestations exist, and even the underlying pathogeneses of these diseases have become somewhat blurred. Clinically, pruritus, as well as other phenotypes of type 2 inflammatory diseases (e.g., elevated IgE and eosinophils), are typical features of AD and yet are observed in 37-75% of patients with psoriasis. Current evidence supports the notion that AD and psoriasis share some clinical and immunopathogenic characteristics based on the following: (1) some patients are genetically susceptible to both diseases; (2) Th17 cells in psoriasis patients may still have the plasticity to re-differentiate toward Th2 phenotypes under certain conditions; (3) clinical treatments can change the manifestations of either disease. To answer whether AD-like psoriasis is a part of a spectrum that may stretch between psoriasis and AD, we must look further at AD patients with symptoms that overlap with those of psoriasis. Here we have conducted a comprehensive literature review to clarify the impact of Th cell plasticity on different immune inflammatory environments, including high levels of IgE in the progression of AD and psoriasis.

## Author contributions

LS and CL: elaborated the figures and wrote the manuscript. LS, HX, and DS: reviewed topics and concepts. DS: conceived, reviewed, and discussed concepts in the manuscript. All authors contributed to critical revisions of the article and approved the submitted version.

## Funding

This work was supported in part by grants from the Traditional Chinese Medicine Science and Technology Program of Shandong Province (NM 2021M080) and the Key Research and Development Plan of Jining (NM 2021YXNS121), Shandong, China.

## Conflict of interest

The authors declare that the research was conducted in the absence of any commercial or financial relationships that could be construed as a potential conflict of interest.

## Publisher's note

All claims expressed in this article are solely those of the authors and do not necessarily represent those of their affiliated organizations, or those of the publisher, the editors and the reviewers. Any product that may be evaluated in this article, or claim that may be made by its manufacturer, is not guaranteed or endorsed by the publisher.

## References

[1] Panaszek B Pawłowicz R Grzegrzółka J Obojski A. Autoreactive IgE in chronic spontaneous/idiopathic urticaria and basophil/mastocyte priming phenomenon, as a feature of autoimmune nature of the syndrome. Arch Immunol Ther Exp (Warsz). (2017) 65:137–43. 10.1007/s00005-016-0417-7 27582030

[2] Zellweger F Eggel A. IgE-associated allergic disorders: recent advances in etiology, diagnosis, and treatment. Allergy. (2016) 71:1652–61. 10.1111/all.13059 27709638

[3] Paparo SB Guaragna MA Albanesi M. High IgE levels in patients affected by psoriasis: review of the literature and personal observations. Clin Ter. (2014) 165:91–3. 10.7471/CT.2014.1682 24770810

[4] Langan SM Irvine AD Weidinger S. Atopic dermatitis. Lancet. (2020) 396:345–60. 10.1016/S0140-6736(20)31286-1 32738956

[5] Griffiths C Armstrong AW Gudjonsson JE Barker J. Psoriasis. Lancet. (2021) 397:1301–15. 10.1016/S0140-6736(20)32549-6 33812489

[6] Furue M Chiba T Tsuji G Ulzii D Kido-Nakahara M Nakahara T. Atopic dermatitis: immune deviation, barrier dysfunction, IgE autoreactivity and new therapies. Allergol Int. (2017) 66:398–403. 10.1016/j.alit.2016.12.002 28057434

[7] Munera-Campos M Carrascosa JM. Innovation in atopic dermatitis: from pathogenesis to treatment. Actas Dermosifiliogr (Engl Ed). (2020) 111:205–21. 10.1016/j.adengl.2020.03.001 31964499

[8] Garcovich S Maurelli M Gisondi P Peris K Yosipovitch G Girolomoni G. Pruritus as a Distinctive Feature of Type 2 Inflammation. Vaccines (Basel). (2021) 9:303. 10.3390/vaccines9030303 33807098 PMC8005108

[9] Jaworecka K Muda-Urban J Rzepko M Reich A. Molecular aspects of pruritus pathogenesis in psoriasis. Int J Mol Sci. (2021) 22:858. 10.3390/ijms22020858 33467067 PMC7830783

[10] Kahremany S Hofmann L Harari M Gruzman A Cohen G. Pruritus in psoriasis and atopic dermatitis: current treatments and new perspectives. Pharmacol Rep. (2021) 73:443–53. 10.1007/s43440-020-00206-y 33460006

[11] Li B Huang L Lv P Li X Liu G Chen Y. The role of Th17 cells in psoriasis. Immunol Res. (2020) 68:296–309. 10.1007/s12026-020-09149-1 32827097

[12] Dai YX Tai YH Chang YT Chen TJ Chen MH. Bidirectional association between psoriasis and atopic dermatitis: a nationwide population-based cohort study. Dermatology. (2021) 237:521–7. 10.1159/000514581 33735855

[13] Boehncke WH Stalder R Brembilla NC. Switching from atopic dermatitis to psoriasis - and back. Rev Med Suisse. (2021) 17:184–7. 10.53738/REVMED.2021.17.723.0184 33507657

[14] Guttman-Yassky E Krueger JG. Atopic dermatitis and psoriasis: two different immune diseases or one spectrum. Curr Opin Immunol. (2017) 48:68–73. 10.1016/j.coi.2017.08.008 28869867

[15] Jensen RK Jabs F Miehe M Mølgaard B Pfützner W Möbs C. Structure of intact IgE and the mechanism of ligelizumab revealed by electron microscopy. Allergy. (2020) 75:1956–65. 10.1111/all.14222 32037590

[16] Godwin L Sinawe H Crane JS. Biochemistry, Immunoglobulin E. Treasure Island, FL: StatPearls (2022). 31082102

[17] Charles N. Autoimmunity, IgE and FcεRI-bearing cells. Curr Opin Immunol. (2021) 72:43–50. 10.1016/j.coi.2021.03.003 33819742

[18] Tanaka S Furuta K. Roles of IgE and Histamine in Mast Cell Maturation. Cells. (2021) 10:2170. 10.3390/cells10082170 34440939 PMC8392195

[19] Gupta RS Warren CM Smith BM Jiang J Blumenstock JA Davis MM. Prevalence and severity of food allergies among US adults. JAMA Netw Open. (2019) 2:e185630. 10.1001/jamanetworkopen.2018.5630 30646188 PMC6324316

[20] Galli SJ. The mast cell-ige paradox: from homeostasis to anaphylaxis. Am J Pathol. (2016) 186:212–24. 10.1016/j.ajpath.2015.07.025 26776074 PMC4729269

[21] Valent P Akin C Hartmann K Nilsson G Reiter A Hermine O. Mast cells as a unique hematopoietic lineage and cell system: From Paul Ehrlich's visions to precision medicine concepts. Theranostics. (2020) 10:10743–68. 10.7150/thno.46719 32929378 PMC7482799

[22] Voehringer D. Protective and pathological roles of mast cells and basophils. Nat Rev Immunol. (2013) 13:362–75. 10.1038/nri3427 23558889

[23] Karasuyama H Miyake K Yoshikawa S Yamanishi Y. Multifaceted roles of basophils in health and disease. J Allergy Clin Immunol. (2018) 142:370–80. 10.1016/j.jaci.2017.10.042 29247714

[24] Voehringer D. Recent advances in understanding basophil functions in vivo. F1000Res. (2017) 6:1464. 10.12688/f1000research.11697.1 28868143 PMC5558098

[25] Schwartz C Turqueti-Neves A Hartmann S Yu P Nimmerjahn F Voehringer D. Basophil-mediated protection against gastrointestinal helminths requires IgE-induced cytokine secretion. Proc Natl Acad Sci U S A. (2014) 111:E5169–77. 10.1073/pnas.1412663111 25404305 PMC4260590

[26] Siracusa MC Saenz SA Hill DA Kim BS Headley MB Doering TA. TSLP promotes interleukin-3-independent basophil haematopoiesis and type 2 inflammation. Nature. (2011) 477:229–33. 10.1038/nature10329 21841801 PMC3263308

[27] Seder RA Paul WE Dvorak AM Sharkis SJ Kagey-Sobotka A Niv Y. Mouse splenic and bone marrow cell populations that express high-affinity Fc epsilon receptors and produce interleukin 4 are highly enriched in basophils. Proc Natl Acad Sci U S A. (1991) 88:2835–9. 10.1073/pnas.88.7.2835 1826367 PMC51334

[28] Gibbs BF Haas H Falcone FH Albrecht C Vollrath IB Noll T. Purified human peripheral blood basophils release interleukin-13 and preformed interleukin-4 following immunological activation. Eur J Immunol. (1996) 26:2493–8. 10.1002/eji.1830261033 8898965

[29] MacGlashan D Jr White JM Huang SK Ono SJ Schroeder JT Lichtenstein LM. Secretion of IL-4 from human basophils. The relationship between IL-4 mRNA and protein in resting and stimulated basophils. J Immunol. (1994) 152:3006–16. 8144899

[30] Mashiko S Bouguermouh S Rubio M Baba N Bissonnette R Sarfati M. Human mast cells are major IL-22 producers in patients with psoriasis and atopic dermatitis. J Allergy Clin Immunol. (2015) 136:351–9.e1. 10.1016/j.jaci.2015.01.033 25792465

[31] Grimbaldeston MA Metz M Yu M Tsai M Galli SJ. Effector and potential immunoregulatory roles of mast cells in IgE-associated acquired immune responses. Curr Opin Immunol. (2006) 18:751–60. 10.1016/j.coi.2006.09.011 17011762

[32] Kawakami T Kitaura J. Mast cell survival and activation by IgE in the absence of antigen: a consideration of the biologic mechanisms and relevance. J Immunol. (2005) 175:4167–73. 10.4049/jimmunol.175.7.4167 16177053 PMC1415266

[33] Kawakami T Galli SJ. Regulation of mast-cell and basophil function and survival by IgE. Nat Rev Immunol. (2002) 2:773–86. 10.1038/nri914 12360215

[34] Kalesnikoff J Huber M Lam V Damen JE Zhang J Siraganian RP. Monomeric IgE stimulates signaling pathways in mast cells that lead to cytokine production and cell survival. Immunity. (2001) 14:801–11. 10.1016/S1074-7613(01)00159-5 11420049

[35] MacGlashan DW Jr Bochner BS Adelman DC Jardieu PM Togias A McKenzie-White J. Down-regulation of Fc(epsilon)RI expression on human basophils during in vivo treatment of atopic patients with anti-IgE antibody. J Immunol. (1997) 158:1438–45. 9013989

[36] Stingl G Maurer D. IgE-mediated allergen presentation via Fc epsilon RI on antigen-presenting cells. Int Arch Allergy Immunol. (1997) 113:24–9. 10.1159/000237499 9130475

[37] Shamji MH Valenta R Jardetzky T Verhasselt V Durham SR Würtzen PA. The role of allergen-specific IgE, IgG and IgA in allergic disease. Allergy. (2021) 76:3627–41. 10.1111/all.14908 33999439 PMC8601105

[38] Eckl-Dorna J Villazala-Merino S Linhart B Karaulov AV Zhernov Y Khaitov M. Allergen-specific antibodies regulate secondary allergen-specific immune responses. Front Immunol. (2018) 9:3131. 10.3389/fimmu.2018.03131 30705676 PMC6344431

[39] Bhoj VG Arhontoulis D Wertheim G Capobianchi J Callahan CA Ellebrecht CT. Persistence of long-lived plasma cells and humoral immunity in individuals responding to CD19-directed CAR T-cell therapy. Blood. (2016) 128:360–70. 10.1182/blood-2016-01-694356 27166358 PMC4957161

[40] Bernasconi NL Traggiai E Lanzavecchia A. Maintenance of serological memory by polyclonal activation of human memory B cells. Science. (2002) 298:2199–202. 10.1126/science.1076071 12481138

[41] Horst A Hunzelmann N Arce S Herber M Manz RA Radbruch A. Detection and characterization of plasma cells in peripheral blood: correlation of IgE+ plasma cell frequency with IgE serum titre. Clin Exp Immunol. (2002) 130:370–8. 10.1046/j.1365-2249.2002.02025.x 12452825 PMC1906552

[42] Eckl-Dorna J Niederberger V. What is the source of serum allergen-specific IgE. Curr Allergy Asthma Rep. (2013) 13:281–7. 10.1007/s11882-013-0348-x 23585215

[43] Chang HD Tokoyoda K Radbruch A. Immunological memories of the bone marrow. Immunol Rev. (2018) 283:86–98. 10.1111/imr.12656 29664564 PMC5947123

[44] Bellou A Kanny G Fremont S Moneret-Vautrin DA. Transfer of atopy following bone marrow transplantation. Ann Allergy Asthma Immunol. (1997) 78:513–6. 10.1016/S1081-1206(10)63240-1 9164366

[45] Brenninkmeijer EE Spuls PI Legierse CM Lindeboom R Smitt JH Bos JD. Clinical differences between atopic and atopiform dermatitis. J Am Acad Dermatol. (2008) 58:407–14. 10.1016/j.jaad.2007.12.002 18280337

[46] Kabashima-Kubo R Nakamura M Sakabe J Sugita K Hino R Mori T. A group of atopic dermatitis without IgE elevation or barrier impairment shows a high Th1 frequency: possible immunological state of the intrinsic type. J Dermatol Sci. (2012) 67:37–43. 10.1016/j.jdermsci.2012.04.004 22591815

[47] Higashi N Niimi Y Aoki M Kawana S. Clinical features of antinuclear antibody-positive patients with atopic dermatitis. J Nippon Med Sch. (2009) 76:300–7. 10.1272/jnms.76.300 20035096

[48] Altrichter S Kriehuber E Moser J Valenta R Kopp T Stingl G. Serum IgE autoantibodies target keratinocytes in patients with atopic dermatitis. J Invest Dermatol. (2008) 128:2232–9. 10.1038/jid.2008.80 18480840

[49] Zeller S Rhyner C Meyer N Schmid-Grendelmeier P Akdis CA Crameri R. Exploring the repertoire of IgE-binding self-antigens associated with atopic eczema. J Allergy Clin Immunol. (2009) 124:278–85, 285.e1–7. 10.1016/j.jaci.2009.05.015 19541355

[50] Kasumagic-Halilovic E. Total serum immunoglobulin E levels in patients with psoriasis. Mater Sociomed. (2020) 32:105–7. 10.5455/msm.2020.32.105-107 32843856 PMC7428889

[51] Al-Janabi A Foulkes AC Griffiths C Warren RB. Paradoxical eczema in patients with psoriasis receiving biologics: a case series. Clin Exp Dermatol. (2022) 47:1174–8. 10.1111/ced.15130 35150003 PMC9310746

[52] Saraceno R Scotto G Chiricozzi A Chimenti S. Urticaria associated with hyper-IgE in a patient with psoriasis undergoing treatment with efalizumab. Acta Derm Venereol. (2009) 89:412–3. 10.2340/00015555-0613 19688158

[53] Yang G Seok JK Kang HC Cho YY Lee HS Lee JY. Skin barrier abnormalities and immune dysfunction in atopic dermatitis. Int J Mol Sci. (2020) 21:2867. 10.3390/ijms21082867 32326002 PMC7215310

[54] Drislane C Irvine AD. The role of filaggrin in atopic dermatitis and allergic disease. Ann Allergy Asthma Immunol. (2020) 124:36–43. 10.1016/j.anai.2019.10.008 31622670

[55] Brunner PM Guttman-Yassky E. Racial differences in atopic dermatitis. Ann Allergy Asthma Immunol. (2019) 122:449–55. 10.1016/j.anai.2018.11.015 30465859

[56] Hertz A Azulay-Abulafia L Nascimento A Ohara CY Kuschnir FC Porto LC. Analysis of filaggrin 2 gene polymorphisms in patients with atopic dermatitis. An Bras Dermatol. (2020) 95:173–9. 10.1016/j.abd.2019.07.002 32151410 PMC7175100

[57] Tokura Y Hayano S. Subtypes of atopic dermatitis: From phenotype to endotype. Allergol Int. (2022) 71:14–24. 10.1016/j.alit.2021.07.003 34344611

[58] David Boothe W Tarbox JA Tarbox MB. Atopic dermatitis: pathophysiology. Adv Exp Med Biol. (2017) 1027:21–37. 10.1007/978-3-319-64804-0_3 29063428

[59] Huang E Ong PY. Severe Atopic Dermatitis in Children. Curr Allergy Asthma Rep. (2018) 18:35. 10.1007/s11882-018-0788-4 29748919

[60] Furue M. Regulation of filaggrin, loricrin, and Involucrin by IL-4, IL-13, IL-17A, IL-22, AHR, and NRF2: pathogenic implications in atopic dermatitis. Int J Mol Sci. (2020) 21:5382. 10.3390/ijms21155382 32751111 PMC7432778

[61] Simpson A Brough HA Haider S Belgrave D Murray CS Custovic A. Early-life inhalant allergen exposure, filaggrin genotype, and the development of sensitization from infancy to adolescence. J Allergy Clin Immunol. (2020) 145:993–1001. 10.1016/j.jaci.2019.08.041 31629803 PMC7057264

[62] Czarnowicki T Krueger JG Guttman-Yassky E. Novel concepts of prevention and treatment of atopic dermatitis through barrier and immune manipulations with implications for the atopic march. J Allergy Clin Immunol. (2017) 139:1723–34. 10.1016/j.jaci.2017.04.004 28583445

[63] Badloe F De Vriese S Coolens K Schmidt-Weber CB Ring J Gutermuth J. IgE autoantibodies and autoreactive T cells and their role in children and adults with atopic dermatitis. Clin Transl Allergy. (2020) 10:34. 10.1186/s13601-020-00338-7 32774842 PMC7398196

[64] Ivert LU Wahlgren CF Lindelöf B Dal H Bradley M Johansson EK. Association between atopic dermatitis and autoimmune diseases: a population-based case-control study. Br J Dermatol. (2021) 185:335–42. 10.1111/bjd.19624 33091150 PMC8451742

[65] Silverberg JI. Association of atopic dermatitis and autoimmune comorbidities: is it real. Br J Dermatol. (2021) 185:243–4. 10.1111/bjd.20497 34121183

[66] Raharja A Mahil SK Barker JN. Psoriasis: a brief overview. Clin Med (Lond). (2021) 21:170–3. 10.7861/clinmed.2021-0257 34001566 PMC8140694

[67] de Alcantara CC Reiche E Simão A. Cytokines in psoriasis. Adv Clin Chem. (2021) 100:171–204. 10.1016/bs.acc.2020.04.004 33453865

[68] Furue M Furue K Tsuji G Nakahara T. Interleukin-17A and Keratinocytes in Psoriasis. Int J Mol Sci. (2020) 21:1275. 10.3390/ijms21041275 32070069 PMC7072868

[69] Rendon A Schäkel K. Psoriasis pathogenesis and treatment. Int J Mol Sci. (2019) 20:1475. 10.3390/ijms20061475 30909615 PMC6471628

[70] Ly K Smith MP Thibodeaux Q Reddy V Liao W Bhutani T. Anti IL-17 in psoriasis. Expert Rev Clin Immunol. (2019) 15:1185–94. 10.1080/1744666X.2020.1679625 31603358

[71] Nickoloff BJ Turka LA. Immunological functions of non-professional antigen-presenting cells: new insights from studies of T-cell interactions with keratinocytes. Immunol Today. (1994) 15:464–9. 10.1016/0167-5699(94)90190-2 7945770

[72] Gillitzer R Wolff K Tong D Müller C Yoshimura T Hartmann AA. MCP-1 mRNA expression in basal keratinocytes of psoriatic lesions. J Invest Dermatol. (1993) 101:127–31. 10.1111/1523-1747.ep12363613 8345212

[73] Bonifacio KM Kunjravia N Krueger JG Fuentes-Duculan J. Cutaneous expression of a disintegrin-like and metalloprotease domain containing thrombospondin type 1 motif-like 5 (ADAMTSL5) in psoriasis goes beyond melanocytes. J Pigment Disord. (2016) 3:244. 10.4172/2376-0427.1000244 27857980 PMC5110039

[74] Nussbaum L Chen YL Ogg GS. Role of regulatory T cells in psoriasis pathogenesis and treatment. Br J Dermatol. (2021) 184:14–24. 10.1111/bjd.19380 32628773

[75] Herster F Bittner Z Archer NK Dickhöfer S Eisel D Eigenbrod T. Neutrophil extracellular trap-associated RNA and LL37 enable self-amplifying inflammation in psoriasis. Nat Commun. (2020) 11:105. 10.1038/s41467-019-13756-4 31913271 PMC6949246

[76] Kaufman BP Alexis AF. Psoriasis in skin of color: insights into the epidemiology, clinical presentation, genetics, quality-of-life impact, and treatment of psoriasis in non-white racial/ethnic groups. Am J Clin Dermatol. (2018) 19:405–23. 10.1007/s40257-017-0332-7 29209945

[77] Nicholas MN Chan AR Hessami-Booshehri M. Psoriasis in patients of color: differences in morphology, clinical presentation, and treatment. Cutis. (2020) 106:7–10;E10. 10.12788/cutis.0038 33104098

[78] Essl A Loader D Feldmann R Steiner A Sator P. Psoriasis and IgE-mediated allergy: correlation or mutual inhibition? A prospective cohort study in patients with mild or moderate to severe psoriasis. Wien Klin Wochenschr. (2021) 133:997–1003. 10.1007/s00508-020-01683-0 32700084

[79] Pigatto PD. Atopy and contact sensitization in psoriasis. Acta Derm Venereol Suppl (Stockh). (2000) 19–20. 10.1080/00015550050500077 11234558

[80] Galili E Barzilai A Twig G Caspi T Daniely D Shreberk-Hassidim R. Allergic rhinitis and asthma among adolescents with psoriasis: a population-based cross-sectional study. Acta Derm Venereol. (2020) 100:adv00133. 10.2340/00015555-3485 32314795 PMC9137373

[81] Ovcina-Kurtovic N Kasumagic-Halilovic E. Serum levels of total immunoglobulin E in patients with psoriasis: relationship with clinical type of disease. Med Arh. (2010) 64:28–9. 20422821

[82] Ding Y Yi X Yu N. Serum IgE levels are increased in patients with generalized pustular psoriasis. Clin Exp Dermatol. (2013) 38:549–52. 10.1111/ced.12086 23777497

[83] Li LF Sujan SA Yang H Wang WH. Serum immunoglobulins in psoriatic erythroderma. Clin Exp Dermatol. (2005) 30:125–7. 10.1111/j.1365-2230.2004.01717.x 15725235

[84] Voulgari PV Gaitanis G Fidhi L Migkos MP Bassukas ID. Joint complaints correlate with increased serum IgE levels in patients hospitalized for moderate-to-severe psoriasis: a single center retrospective study. J Am Acad Dermatol. (2016) 74:1014–5.e4. 10.1016/j.jaad.2015.11.026 27085234

[85] Watts MM Marie Ditto A. Anaphylaxis. Allergy Asthma Proc. (2019) 40:453–6. 10.2500/aap.2019.40.4270 31690393

[86] Yan KX Huang Q Fang X Zhang ZH Han L Gadaldi K. IgE and FcεRI are highly expressed on innate cells in psoriasis. Br J Dermatol. (2016) 175:122–33. 10.1111/bjd.14459 26853903

[87] Hajdarbegovic E Nijsten T Westgeest A Habraken F Hollestein L Thio B. Decreased prevalence of atopic features in patients with psoriatic arthritis, but not in psoriasis vulgaris. J Am Acad Dermatol. (2013) 68:270–7. 10.1016/j.jaad.2012.07.018 22921106

[88] Armas-González E Díaz-Martín A Domínguez-Luis MJ Arce-Franco MT Herrera-García A Hernández-Hernández MV. Differential antigen-presenting B cell phenotypes from synovial microenvironment of patients with rheumatoid and psoriatic arthritis. J Rheumatol. (2015) 42:1825–34. 10.3899/jrheum.141577 26178284

[89] Haniuda K Kitamura D. Multi-faceted regulation of IgE production and humoral memory formation. Allergol Int. (2021) 70:163–8. 10.1016/j.alit.2020.11.002 33288436

[90] Grund LZ Komegae EN Lopes-Ferreira M Lima C. IL-5 and IL-17A are critical for the chronic IgE response and differentiation of long-lived antibody-secreting cells in inflamed tissues. Cytokine. (2012) 59:335–51. 10.1016/j.cyto.2012.04.045 22633287

[91] Furue M Ulzii D Vu YH Tsuji G Kido-Nakahara M Nakahara T. Pathogenesis of atopic dermatitis: current paradigm. Iran J Immunol. (2019) 16:97–107. 31182684 10.22034/IJI.2019.80253

[92] Milovanovic M Drozdenko G Weise C Babina M Worm M. Interleukin-17A promotes IgE production in human B cells. J Invest Dermatol. (2010) 130:2621–8. 10.1038/jid.2010.175 20596087

[93] Kobayashi S Haruo N Sugane K Ochs HD Agematsu K. Interleukin-21 stimulates B-cell immunoglobulin E synthesis in human beings concomitantly with activation-induced cytidine deaminase expression and differentiation into plasma cells. Hum Immunol. (2009) 70:35–40. 10.1016/j.humimm.2008.10.021 19026702

[94] Hong GU Park BS Park JW Kim SY Ro JY. IgE production in CD40/CD40L cross-talk of B and mast cells and mediator release *via* TGase 2 in mouse allergic asthma. Cell Signal. (2013) 25:1514–25. 10.1016/j.cellsig.2013.03.010 23524335

[95] Ishiuji Y Umezawa Y Asahina A Fukuta H Aizawa N Yanaba K. Exacerbation of atopic dermatitis symptoms by ustekinumab in psoriatic patients with elevated serum immunoglobulin E levels: Report of two cases. J Dermatol. (2018) 45:732–4. 10.1111/1346-8138.14295 29569296

[96] Lis-Swiety A Skrzypek-Salamon A Arasiewicz H Brzezińska-Wcisło L. Atopic dermatitis exacerbated with ustekinumab in a psoriatic patient with childhood history of atopy. Allergol Int. (2015) 64:382–3. 10.1016/j.alit.2015.06.003 26433537

[97] Sugiura R Terui H Shimada-Omori R Yamazaki E Tsuchiyama K Takahashi T. Biologics modulate antinuclear antibodies, immunoglobulin E, and eosinophil counts in psoriasis patients. J Dermatol. (2021) 48:1739–44. 10.1111/1346-8138.16102 34368997

[98] Ding F Fu Z Liu B. Lipopolysaccharide exposure alleviates asthma in mice by regulating Th1/Th2 and Treg/Th17 balance. Med Sci Monit. (2018) 24:3220–9. 10.12659/MSM.905202 29768397 PMC5985709

[99] Beer WE Smith AE Kassab JY Smith PH Rowland Payne CM. Concomitance of psoriasis and atopic dermatitis. Dermatology. (1992) 184:265–70. 10.1159/000247564 1482441

[100] Nanda A. Concomitance of psoriasis and atopic dermatitis. Dermatology. (1995) 191:72. 10.1159/000246495 8589492

[101] Barry K Zancanaro P Casseres R Abdat R Dumont N Rosmarin D. Concomitant atopic dermatitis and psoriasis - a retrospective review. J Dermatolog Treat. (2021) 32:716–20. 10.1080/09546634.2019.1702147 31801394

[102] García-Souto F Lorente-Lavirgen AI Bernabéu-Wittel J Rojas C Lorente R. Long-lasting contact dermatitis in patients with atopic dermatitis or psoriasis. Australas J Dermatol. (2020) 61:342–5. 10.1111/ajd.13367 32662093

[103] Silverberg JI Hou A DeKoven JG Warshaw EM Maibach HI Atwater AR. Prevalence and trend of allergen sensitization in patients referred for patch testing with a final diagnosis of psoriasis: North American Contact Dermatitis Group data, 2001-2016. Contact Dermatitis. (2021) 85:435–45. 10.1111/cod.13877 33931870

[104] Al-Janabi A Foulkes AC Mason K Smith CH Griffiths C Warren RB. Phenotypic switch to eczema in patients receiving biologics for plaque psoriasis: a systematic review. J Eur Acad Dermatol Venereol. (2020) 34:1440–8. 10.1111/jdv.16246 31997406

[105] Devos M Mogilenko DA Fleury S Gilbert B Becquart C Quemener S. Keratinocyte expression of A20/TNFAIP3 controls skin inflammation associated with atopic dermatitis and psoriasis. J Invest Dermatol. (2019) 139:135–45. 10.1016/j.jid.2018.06.191 30118730

[106] Bowcock AM Cookson WO. The genetics of psoriasis, psoriatic arthritis and atopic dermatitis. Hum Mol Genet. (2004) 13:R43-55. 10.1093/hmg/ddh094 14996755

[107] Cookson WO Ubhi B Lawrence R Abecasis GR Walley AJ Cox HE. Genetic linkage of childhood atopic dermatitis to psoriasis susceptibility loci. Nat Genet. (2001) 27:372–3. 10.1038/86867 11279517

[108] Tamari M Saeki H Hayashi M Umezawa Y Ito T Fukuchi O. An association study of 36 psoriasis susceptibility loci for psoriasis vulgaris and atopic dermatitis in a Japanese population. J Dermatol Sci. (2014) 76:156–7. 10.1016/j.jdermsci.2014.08.005 25205357

[109] Baurecht H Hotze M Brand S Büning C Cormican P Corvin A. Genome-wide comparative analysis of atopic dermatitis and psoriasis gives insight into opposing genetic mechanisms. Am J Hum Genet. (2015) 96:104–20. 10.1016/j.ajhg.2014.12.004 25574825 PMC4289690

[110] Brown SJ Asai Y Cordell HJ Campbell LE Zhao Y Liao H. Loss-of-function variants in the filaggrin gene are a significant risk factor for peanut allergy. J Allergy Clin Immunol. (2011) 127:661–7. 10.1016/j.jaci.2011.01.031 21377035 PMC3081065

[111] Chang YC Wu WM Chen CH Hu CF Hsu LA. Association between P478S polymorphism of the filaggrin gene and risk of psoriasis in a Chinese population in Taiwan. Arch Dermatol Res. (2008) 300:133–7. 10.1007/s00403-007-0821-2 18193244

[112] Wongpiyabovorn J Suto H Ushio H Izuhara K Mitsuishi K Ikeda S. Up-regulation of interleukin-13 receptor alpha1 on human keratinocytes in the skin of psoriasis and atopic dermatitis. J Dermatol Sci. (2003) 33:31–40. 10.1016/S0923-1811(03)00148-8 14527737

[113] Bowes J Eyre S Flynn E Ho P Salah S Warren RB. Evidence to support IL-13 as a risk locus for psoriatic arthritis but not psoriasis vulgaris. Ann Rheum Dis. (2011) 70:1016–9. 10.1136/ard.2010.143123 21349879 PMC3086035

[114] Chan AR Sandhu VK Drucker AM Fleming P Lynde CW. Adult-onset atopic dermatitis: presentations and progress. J Cutan Med Surg. (2020) 24:267–72. 10.1177/1203475420911896 32238071

[115] Silverberg JI Vakharia PP Chopra R Sacotte R Patel N Immaneni S. Phenotypical differences of childhood- and adult-onset atopic dermatitis. J Allergy Clin Immunol Pract. (2018) 6:1306–12. 10.1016/j.jaip.2017.10.005 29133223 PMC5945342

[116] Furue M Kadono T. “Inflammatory skin march” in atopic dermatitis and psoriasis. Inflamm Res. (2017) 66:833–42. 10.1007/s00011-017-1065-z 28620798

[117] Feramisco JD Berger TG Steinhoff M. Innovative management of pruritus. Dermatol Clin. (2010) 28:467–78. 10.1016/j.det.2010.03.004 20510757

[118] Yosipovitch G Goon AT Wee J Chan YH Zucker I Goh CL. Itch characteristics in Chinese patients with atopic dermatitis using a new questionnaire for the assessment of pruritus. Int J Dermatol. (2002) 41:212–6. 10.1046/j.1365-4362.2002.01460.x 12031029

[119] Reich A Szepietowski JC. Clinical Aspects of Itch: Psoriasis. Boca Raton, FL: CRC Press/Taylor & Francis (2014). 24830002

[120] Nattkemper LA Tey HL Valdes-Rodriguez R Lee H Mollanazar NK Albornoz C. The genetics of chronic itch: gene expression in the skin of patients with atopic dermatitis and psoriasis with severe itch. J Invest Dermatol. (2018) 138:1311–7. 10.1016/j.jid.2017.12.029 29317264

[121] Kulthanan K Boochangkool K Tuchinda P Chularojanamontri L. Clinical features of the extrinsic and intrinsic types of adult-onset atopic dermatitis. Asia Pac Allergy. (2011) 1:80–6. 10.5415/apallergy.2011.1.2.80 22053301 PMC3206251

[122] Suárez-Fariñas M Dhingra N Gittler J Shemer A Cardinale I de Guzman Strong C. Intrinsic atopic dermatitis shows similar TH2 and higher TH17 immune activation compared with extrinsic atopic dermatitis. J Allergy Clin Immunol. (2013) 132:361–70. 10.1016/j.jaci.2013.04.046 23777851 PMC3991240

[123] Tanei R Hasegawa Y. Atopic dermatitis in older adults: a viewpoint from geriatric dermatology. Geriatr Gerontol Int. (2016) 16 Suppl 1:75–86. 10.1111/ggi.12771 27018286

[124] Nomura T Wu J Kabashima K Guttman-Yassky E. Endophenotypic variations of atopic dermatitis by age, race, and ethnicity. J Allergy Clin Immunol Pract. (2020) 8:1840–52. 10.1016/j.jaip.2020.02.022 32499033

[125] Renert-Yuval Y Del Duca E Pavel AB Fang M Lefferdink R Wu J. The molecular features of normal and atopic dermatitis skin in infants, children, adolescents, and adults. J Allergy Clin Immunol. (2021) 148:148–63. 10.1016/j.jaci.2021.01.001 33453290 PMC9285652

[126] Czarnowicki T He H Canter T Han J Lefferdink R Erickson T. Evolution of pathologic T-cell subsets in patients with atopic dermatitis from infancy to adulthood. J Allergy Clin Immunol. (2020) 145:215–28. 10.1016/j.jaci.2019.09.031 31626841 PMC6957229

[127] Leung DY. Atopic dermatitis: age and race do matter. J Allergy Clin Immunol. (2015) 136:1265–7. 10.1016/j.jaci.2015.09.011 26549637

[128] Kaufman BP Guttman-Yassky E Alexis AF. Atopic dermatitis in diverse racial and ethnic groups-Variations in epidemiology, genetics, clinical presentation and treatment. Exp Dermatol. (2018) 27:340–57. 10.1111/exd.13514 29457272

[129] Noda S Suárez-Fariñas M Ungar B Kim SJ de Guzman Strong C Xu H. The Asian atopic dermatitis phenotype combines features of atopic dermatitis and psoriasis with increased TH17 polarization. J Allergy Clin Immunol. (2015) 136:1254–64. 10.1016/j.jaci.2015.08.015 26428954

[130] van der Fits L Mourits S Voerman JS Kant M Boon L Laman JD. Imiquimod-induced psoriasis-like skin inflammation in mice is mediated via the IL-23/IL-17 axis. J Immunol. (2009) 182:5836–45. 10.4049/jimmunol.0802999 19380832

[131] Okada K Matsushima Y Mizutani K Yamanaka K. The Role of Gut Microbiome in Psoriasis: Oral Administration of Staphylococcus aureus and Streptococcus danieliae Exacerbates Skin Inflammation of Imiquimod-Induced Psoriasis-Like Dermatitis. Int J Mol Sci. (2020) 21:3303. 10.3390/ijms21093303 32392785 PMC7246652

[132] Couderc E Morel F Levillain P Buffière-Morgado A Camus M Paquier C. Interleukin-17A-induced production of acute serum amyloid A by keratinocytes contributes to psoriasis pathogenesis. PLoS ONE. (2017) 12:e0181486. 10.1371/journal.pone.0181486 28708859 PMC5510841

[133] Kamijo S Hara M Suzuki M Nakae S Ogawa H Okumura K. Innate IL-17A enhances IL-33-independent skin eosinophilia and IgE response on subcutaneous papain sensitization. J Invest Dermatol. (2021) 141:105–13.e14. 10.1016/j.jid.2020.05.088 32470341

[134] Zbikowska-Gotz M Pałgan K Gawrońska-Ukleja E Kuzmiński A Przybyszewski M Socha E. Expression of IL-17A concentration and effector functions of peripheral blood neutrophils in food allergy hypersensitivity patients. Int J Immunopathol Pharmacol. (2016) 29:90–8. 10.1177/0394632015617069 26684636 PMC5806745

[135] Hofmann MA Fluhr JW Ruwwe-Glösenkamp C Stevanovic K Bergmann KC Zuberbier T. Role of IL-17 in atopy-A systematic review. Clin Transl Allergy. (2021) 11:e12047. 10.1002/clt2.12047 34429872 PMC8361814

[136] Chen J Li C Li H Yu H Zhang X Yan M. Identification of a T(H) 2-high psoriasis cluster based on skin biomarker analysis in a Chinese psoriasis population. J Eur Acad Dermatol Venereol. (2021) 35:150–8. 10.1111/jdv.16563 32367566

[137] Brembilla NC Senra L Boehncke WH. The IL-17 family of cytokines in psoriasis: IL-17A and Beyond. Front Immunol. (2018) 9:1682. 10.3389/fimmu.2018.01682 30127781 PMC6088173

[138] Cataldi C Mari NL Lozovoy M Martins L Reiche E Maes M. Proinflammatory and anti-inflammatory cytokine profiles in psoriasis: use as laboratory biomarkers and disease predictors. Inflamm Res. (2019) 68:557–67. 10.1007/s00011-019-01238-8 31062065

[139] Cosmi L Maggi L Santarlasci V Capone M Cardilicchia E Frosali F. Identification of a novel subset of human circulating memory CD4(+) T cells that produce both IL-17A and IL-4. J Allergy Clin Immunol. (2010) 125:222–30.e1-4. 10.1016/j.jaci.2009.10.012 20109749

[140] Cosmi L Santarlasci V Maggi L Liotta F Annunziato F. Th17 plasticity: pathophysiology and treatment of chronic inflammatory disorders. Curr Opin Pharmacol. (2014) 17:12–6. 10.1016/j.coph.2014.06.004 24980083

[141] Lozano-Ojalvo D Tyler SR Berin MC. Is the plasticity of the Th17 subset a key source of allergenic Th2 responses. Allergy. (2021) 76:3238–40. 10.1111/all.14888 33930200

[142] Elango T Sun J Zhu C Zhou F Zhang Y Sun L. Mutational analysis of epidermal and hyperproliferative type I keratins in mild and moderate psoriasis vulgaris patients: a possible role in the pathogenesis of psoriasis along with disease severity. Hum Genomics. (2018) 12:27. 10.1186/s40246-018-0158-2 29784039 PMC5963134

[143] Akhlaghi M Karrabi M Atabti H Raoofi A Mousavi Khaneghah A. Investigation of the role of IL18, IL-1β and NLRP3 inflammasome in reducing expression of FLG-2 protein in Psoriasis vulgaris skin lesions. Biotech Histochem. (2022) 97:277–83. 10.1080/10520295.2021.1954692 34313166

[144] Kim BE Howell MD Guttman-Yassky E Gilleaudeau PM Cardinale IR Boguniewicz M. TNF-α downregulates filaggrin and loricrin through c-Jun N-terminal kinase: role for TNF-α antagonists to improve skin barrier. J Invest Dermatol. (2011) 131:1272–9. 10.1038/jid.2011.24 21346775 PMC8609659

[145] Kirschner N Poetzl C von den Driesch P Wladykowski E Moll I Behne MJ. Alteration of tight junction proteins is an early event in psoriasis: putative involvement of proinflammatory cytokines. Am J Pathol. (2009) 175:1095–106. 10.2353/ajpath.2009.080973 19661441 PMC2731128

[146] Chung E Cook PW Parkos CA Park YK Pittelkow MR Coffey RJ. Amphiregulin causes functional downregulation of adherens junctions in psoriasis. J Invest Dermatol. (2005) 124:1134–40. 10.1111/j.0022-202X.2005.23762.x 15955087

[147] Labarthe MP Bosco D Saurat JH Meda P Salomon D. Upregulation of connexin 26 between keratinocytes of psoriatic lesions. J Invest Dermatol. (1998) 111:72–6. 10.1046/j.1523-1747.1998.00248.x 9665389

[148] Stylianaki EA Karpouzis A Tripsianis G Veletza S. Assessment of gap junction protein beta-2 rs3751385 gene polymorphism in psoriasis vulgaris. J Clin Med Res. (2019) 11:642–50. 10.14740/jocmr3845 31523338 PMC6731047

[149] Łuczaj W Wroński A Domingues P Domingues MR Skrzydlewska E. Lipidomic analysis reveals specific differences between fibroblast and keratinocyte ceramide profile of patients with psoriasis vulgaris. Molecules. (2020) 25:630. 10.3390/molecules25030630 32023992 PMC7037443

[150] Pietrzak A Michalak-Stoma A Chodorowska G Szepietowski JC. Lipid disturbances in psoriasis: an update. Mediators Inflamm. (2010) 2010:535612. 10.1155/2010/535612 20706605 PMC2914266

[151] Varshney P Narasimhan A Mittal S Malik G Sardana K Saini N. Transcriptome profiling unveils the role of cholesterol in IL-17A signaling in psoriasis. Sci Rep. (2016) 6:19295. 10.1038/srep19295 26781963 PMC4726068

[152] Lee Y Je YJ Lee SS Li ZJ Choi DK Kwon YB. Changes in transepidermal water loss and skin hydration according to expression of aquaporin-3 in psoriasis. Ann Dermatol. (2012) 24:168–74. 10.5021/ad.2012.24.2.168 22577267 PMC3346907

[153] Gegotek A Domingues P Wroński A Skrzydlewska E. Changes in proteome of fibroblasts isolated from psoriatic skin lesions. Int J Mol Sci. (2020) 21:5363. 10.3390/ijms21155363 32731552 PMC7432102

[154] Ten Bergen LL Petrovic A Aarebrot AK Appel S. Current knowledge on autoantigens and autoantibodies in psoriasis. Scand J Immunol. (2020) 92:e12945. 10.1111/sji.12945 32697368

[155] Lou F Sun Y Xu Z Niu L Wang Z Deng S. Excessive polyamine generation in keratinocytes promotes self-RNA sensing by dendritic cells in psoriasis. Immunity. (2020) 53:204–16.e10. 10.1016/j.immuni.2020.06.004 32553276

[156] Takahashi T Yamasaki K. Psoriasis and antimicrobial peptides. Int J Mol Sci. (2020) 21:6791. 10.3390/ijms21186791 32947991 PMC7555190

[157] Albanesi C Madonna S Gisondi P Girolomoni G. The interplay between keratinocytes and immune cells in the pathogenesis of psoriasis. Front Immunol. (2018) 9:1549. 10.3389/fimmu.2018.01549 30034395 PMC6043636

[158] Cai Y Xue F Quan C Qu M Liu N Zhang Y. A Critical role of the IL-1β-IL-1R signaling pathway in skin inflammation and psoriasis pathogenesis. J Invest Dermatol. (2019) 139:146–56. 10.1016/j.jid.2018.07.025 30120937 PMC6392027

[159] Orlik C Deibel D Küblbeck J Balta E Ganskih S Habicht J. Keratinocytes costimulate naive human T cells via CD2: a potential target to prevent the development of proinflammatory Th1 cells in the skin. Cell Mol Immunol. (2020) 17:380–94. 10.1038/s41423-019-0261-x 31324882 PMC7109061

[160] Tohyama M Yang L Hanakawa Y Dai X Shirakata Y Sayama K. IFN-α enhances IL-22 receptor expression in keratinocytes: a possible role in the development of psoriasis. J Invest Dermatol. (2012) 132:1933–5. 10.1038/jid.2011.468 22297633

[161] Chiricozzi A Romanelli P Volpe E Borsellino G Romanelli M. Scanning the immunopathogenesis of psoriasis. Int J Mol Sci. (2018) 19:179. 10.3390/ijms19010179 29316717 PMC5796128

[162] Boonpiyathad T Sözener ZC Satitsuksanoa P Akdis CA. Immunologic mechanisms in asthma. Semin Immunol. (2019) 46:101333. 10.1016/j.smim.2019.101333 31703832

[163] Chiang CC Cheng WJ Korinek M Lin CY Hwang TL. Neutrophils in Psoriasis. Front Immunol. (2019) 10:2376. 10.3389/fimmu.2019.02376 31649677 PMC6794444

[164] Wang Y Edelmayer R Wetter J Salte K Gauvin D Leys L. Monocytes/Macrophages play a pathogenic role in IL-23 mediated psoriasis-like skin inflammation. Sci Rep. (2019) 9:5310. 10.1038/s41598-019-41655-7 30926837 PMC6441056

[165] Choi CW Kim BR Yang S Kim Y Kang JS Youn SW. Regulatory T cells suppress skin inflammation in the imiquimod-induced psoriasis-like mouse model. J Dermatol Sci. (2020) 98:199–202. 10.1016/j.jdermsci.2020.04.008 32451152

[166] Hayashi M Yanaba K Umezawa Y Yoshihara Y Kikuchi S Ishiuji Y. IL-10-producing regulatory B cells are decreased in patients with psoriasis. J Dermatol Sci. (2016) 81:93–100. 10.1016/j.jdermsci.2015.11.003 26614745

[167] Cao LY Chung JS Teshima T Feigenbaum L Cruz PD Jr Jacobe HT. Myeloid-derived suppressor cells in psoriasis are an expanded population exhibiting diverse T-cell-suppressor mechanisms. J Invest Dermatol. (2016) 136:1801–10. 10.1016/j.jid.2016.02.816 27236103 PMC4992618

[168] Chaowattanapanit S Choonhakarn C Salao K Winaikosol K Julanon N Wongjirattikarn R. Increased serum IL-31 levels in chronic spontaneous urticaria and psoriasis with pruritic symptoms. Heliyon. (2020) 6:e05621. 10.1016/j.heliyon.2020.e05621 33305054 PMC7711144

[169] Purzycka-Bohdan D Gleñ J Zabłotna M Nedoszytko B Szczerkowska-Dobosz A Sokołowska-Wojdyło M. Significance of interleukin-31 (IL-31) gene polymorphisms and IL-31 serum level in psoriasis in correlation with pruritus. Postepy Dermatol Alergol. (2021) 38:657–64. 10.5114/ada.2021.108926 34658710 PMC8501425

[170] Elewski B Alexis AF Lebwohl M Stein Gold L Pariser D Del Rosso J. Itch: an under-recognized problem in psoriasis. J Eur Acad Dermatol Venereol. (2019) 33:1465–76. 10.1111/jdv.15450 30680819

[171] Mack MR Kim BS. The itch-scratch cycle: a neuroimmune perspective. Trends Immunol. (2018) 39:980–91. 10.1016/j.it.2018.10.001 30471983 PMC8896504

[172] Mócsai G Gáspár K Dajnoki Z Tóth B Gyimesi E Bíró T. Investigation of skin barrier functions and allergic sensitization in patients with hyper-ige syndrome. J Clin Immunol. (2015) 35:681–8. 10.1007/s10875-015-0200-2 26453584

[173] Xiao C Puddicombe SM Field S Haywood J Broughton-Head V Puxeddu I. Defective epithelial barrier function in asthma. J Allergy Clin Immunol. (2011) 128:549–6.e1-12. 10.1016/j.jaci.2011.05.038 21752437

[174] Irvine AD McLean WH Leung DY. Filaggrin mutations associated with skin and allergic diseases. N Engl J Med. (2011) 365:1315–27. 10.1056/NEJMra1011040 21991953

[175] Georas SN Rezaee F. Epithelial barrier function: at the front line of asthma immunology and allergic airway inflammation. J Allergy Clin Immunol. (2014) 134:509–20. 10.1016/j.jaci.2014.05.049 25085341 PMC4170838

[176] Johansson EK Bergström A Kull I Lind T Söderhäll C van Hage M. IgE sensitization in relation to preschool eczema and filaggrin mutation. J Allergy Clin Immunol. (2017) 140:1572–9.e5. 10.1016/j.jaci.2017.04.008 28456621

[177] Pothoven KL Schleimer RP. The barrier hypothesis and Oncostatin M: Restoration of epithelial barrier function as a novel therapeutic strategy for the treatment of type 2 inflammatory disease. Tissue Barriers. (2017) 5:e1341367. 10.1080/21688370.2017.1341367 28665760 PMC5571776

[178] Luo L Luo Y Xu J Zhu R Wu J Liu X. Heterogeneous origin of IgE in atopic dermatitis and psoriasis revealed by B cell receptor repertoire analysis. Allergy. (2022) 77:559–68. 10.1111/all.15173 34738638

[179] Wu LC Zarrin AA. The production and regulation of IgE by the immune system. Nat Rev Immunol. (2014) 14:247–59. 10.1038/nri3632 24625841

[180] Sukseree S Bakiri L Palomo-Irigoyen M Uluçkan Ö Petzelbauer P Wagner EF. Sequestosome 1/p62 enhances chronic skin inflammation. J Allergy Clin Immunol. (2021) 147:2386–93.e4. 10.1016/j.jaci.2021.02.028 33675820

[181] Jiao Q Qian Q Liu C Luo Y Fang F Wang M. T helper 22 cells from Han Chinese patients with atopic dermatitis exhibit high expression of inducible T-cell costimulator. Br J Dermatol. (2020) 182:648–57. 10.1111/bjd.18040 31090221

[182] Miyagaki T Sugaya M. Recent advances in atopic dermatitis and psoriasis: genetic background, barrier function, and therapeutic targets. J Dermatol Sci. (2015) 78:89–94. 10.1016/j.jdermsci.2015.02.010 25771165

[183] Shin D Kim DS Kim SH Je JH Kim HJ Young Kim D. Decreased PD-1 positive blood follicular helper T cells in patients with psoriasis. Arch Dermatol Res. (2016) 308:593–9. 10.1007/s00403-016-1679-y 27501809

[184] Lombardi V Singh AK Akbari O. The role of costimulatory molecules in allergic disease and asthma. Int Arch Allergy Immunol. (2010) 151:179–89. 10.1159/000242355 19786798 PMC2837887

[185] He H Bissonnette R Wu J Diaz A Saint-Cyr Proulx E Maari C. Tape strips detect distinct immune and barrier profiles in atopic dermatitis and psoriasis. J Allergy Clin Immunol. (2021) 147:199–212. 10.1016/j.jaci.2020.05.048 32709423

[186] Vercelli D Jabara HH Arai K Geha RS. Induction of human IgE synthesis requires interleukin 4 and T/B cell interactions involving the T cell receptor/CD3 complex and MHC class II antigens. J Exp Med. (1989) 169:1295–307. 10.1084/jem.169.4.1295 2522501 PMC2189234

[187] Krzyzak L Seitz C Urbat A Hutzler S Ostalecki C Gläsner J. CD83 Modulates B cell activation and germinal center responses. J Immunol. (2016) 196:3581–94. 10.4049/jimmunol.1502163 26983787

[188] Matucci A Vultaggio A Maggi E Kasujee I. Is IgE or eosinophils the key player in allergic asthma pathogenesis? Are we asking the right question. Respir Res. (2018) 19:113. 10.1186/s12931-018-0813-0 29879991 PMC5992661

[189] Butcher MJ Zhu J. Recent advances in understanding the Th1/Th2 effector choice. Fac Rev. (2021) 10:30. 10.12703/r/10-30 33817699 PMC8009194

[190] Asayama K Kobayashi T D' Alessandro-Gabazza CN Toda M Yasuma T Fujimoto H. Protein S protects against allergic bronchial asthma by modulating Th1/Th2 balance. Allergy. (2020) 75:2267–78. 10.1111/all.14261 32145080

[191] Heeringa JJ van Zelm MC. Is there a pathogenic role for IgE in psoriasis. Br J Dermatol. (2016) 175:16–7. 10.1111/bjd.14607 27484269

[192] Wollenberg A Thomsen SF Lacour JP Jaumont X Lazarewicz S. Targeting immunoglobulin E in atopic dermatitis: a review of the existing evidence. World Allergy Organ J. (2021) 14:100519. 10.1016/j.waojou.2021.100519 33815652 PMC8005850

[193] Tang TS Bieber T Williams HC. Does “autoreactivity” play a role in atopic dermatitis. J Allergy Clin Immunol. (2012) 129:1209–15.e2. 10.1016/j.jaci.2012.02.002 22409986

[194] Roesner LM Werfel T. Autoimmunity (or Not) in atopic dermatitis. Front Immunol. (2019) 10:2128. 10.3389/fimmu.2019.02128 31552053 PMC6746887

[195] Holmes J Fairclough LC Todd I. Atopic dermatitis and autoimmunity: the occurrence of autoantibodies and their association with disease severity. Arch Dermatol Res. (2019) 311:141–62. 10.1007/s00403-019-01890-4 30798353 PMC7192884

